# Development of SSR Markers and Genetic Diversity Analysis of *Musa basjoo* Using Transcriptome Sequencing

**DOI:** 10.1002/ece3.72128

**Published:** 2025-09-10

**Authors:** Jian Zhang, Chenglong Yang, Xiaoyu Yang, Jing Lei, Peiling Long

**Affiliations:** ^1^ GuiZhou Institute of Subtropical Crops Guizhou Academy of Agricultural Sciences Guiyang China; ^2^ Ministry of Agriculture and Rural Affairs Key Laboratory of Crop Genetic Resources and Germplasm Innovation in Karst Region Guiyang China

**Keywords:** genetic diversity, *M. basjoo*, SSR markers, transcriptome

## Abstract

*Rhizoma Musae*, the underground rhizome of *Musa basjoo* Siebold et Zucc., serves as a traditional medicinal resource among the Miao ethnic group in Guizhou, China. This study aimed to develop SSR molecular markers from the *M. basjoo* transcriptome to assess the genetic diversity of its germplasm across diverse regions. A *M. basjoo* transcriptome database was constructed using high‐throughput sequencing, followed by the extraction of SSR loci with MISA software. Polymorphic SSR markers were identified and utilized to evaluate the genetic diversity of 45 *M. basjoo* germplasm resources. In total, 38,806 *M. basjoo* Unigenes were obtained, with an average length of 993.00 bp. Comparative annotation against the GO, KEGG, EggNOG, NR, Swiss‐prot, and Pfam databases yielded 23,558, 12,412, 25,448, 28,101, 20,056, and 18,460 annotated Unigenes, respectively. A total of 7501 SSR loci were identified, with trinucleotide repeats constituting the predominant class (44.03%), and ACC/GGT being the most frequent motif. Ten pairs of polymorphic primers were developed, all exhibiting a PIC value greater than 0.5. The 45 *Musa* spp. accessions were classified into three distinct groups using the UPGMA method. The *M. basjoo* transcriptome harbors abundant polymorphic SSRs. The developed primers enable rapid molecular identification of *M. basjoo* germplasm and provide insights into its genetic diversity, which may underlie variations in medicinal properties and environmental adaptation. These findings support applications in germplasm conservation, marker‐assisted breeding, and quality control of *Rhizoma Musae* medicinal resources.

## Abstract 2

We designed 100 pairs of specific primers on the basis of the characteristics of the transcriptome SSR loci of *Rhizoma Musae*. After the final screening, 10 pairs of polymorphic primers were determined. The PIC values of the selected 10 pairs of polymorphic primers are all greater than 0.5, and they can be used for cluster analysis of 45 *Rhizoma Musae* resources. This provides an important reference for the molecular characterization and identification of *Musa basjoo* roots.

## 1 Introduction

*Musa* spp., a widely distributed tropical and subtropical plant (Chen et al. 2017), thrives in southern China, including Guangdong, Hainan, Guizhou, and other regions. *Rhizoma Musae* refers to the dried rhizome of *Musa basjoo* (Musaceae). It is a traditional medicinal resource among the Miao ethnic group in Guizhou Province, China, and is listed in the *Quality Standards for Traditional Chinese and Ethnic Medicinal Materials in Guizhou Province (2019 edition)* (Guizhou Provincial Drug Administration 2020). Known for its anti‐inflammatory (Zhang et al. 2025a), analgesic (Liang et al. 2017), antibacterial (Wei et al. 2010), and antioxidant (Liu et al. 2013) properties, *Rhizoma Musae* has been the subject of numerous pharmacological studies. These studies have identified a wide range of bioactive compounds, including sugars, amino acids, organic acids, flavonoids, steroids, and volatile oils (Li et al. 2023). Notably, Gukang Capsule, which incorporates *Rhizoma Musae* as its primary ingredient, has demonstrated significant clinical efficacy in treating osteoarthritis, fractures, and related conditions (Zhu et al. 2022).

Simple sequence repeats (SSRs), or microsatellites with tandem repeat sequences of 1 to 6 nucleotides as basic motifs, are widespread throughout the genome and exhibit high polymorphism and codominant inheritance, making them effective markers for assessing genetic variation across individuals and populations (Zhuo et al. 2021). Recent advancements in sequencing technologies and the availability of transcriptome data have further heightened their application in the molecular biology of medicinal plants. Because of their extensive distribution, expressed sequence tag‐simple sequence repeats (EST‐SSR) have become a valuable tool for evaluating species genetic diversity (Dai et al. 2017), particularly in cases where reference genomes are unavailable, such as *Fructus amomi* (Li et al. 2022), *Atractylodes lancea* (Ma et al. 2022), and *Asarum sieboldii* (Chen et al. 2023). Currently, the majority of *Rhizoma Musae* medicinal materials are sourced from wild collections. However, because of the morphological similarities among *Musa* spp. plants, accurate identification via traditional morphological methods remains challenging. Additionally, artificial cultivation of *Rhizoma Musae* is still in its early stages, and the genetic relationships between germplasm resources from different origins are not yet well understood, which significantly hampers efforts to analyze and utilize these resources effectively.

In this study, transcriptome sequencing of *M. basjoo* was employed to analyze the composition, distribution, and characteristics of EST‐SSR loci in *M. basjoo*. Polymorphic EST‐SSR markers were developed, enabling the genetic classification of *M. basjoo* resources with varying population structures. This study's results establish a solid foundation for *Musa basjoo* germplasm conservation, marker‐assisted breeding, and analysis of medicinal varieties across different production regions. The newly developed SSR markers facilitate accurate identification of *M. basjoo* and differentiation from related species, thereby safeguarding precious genetic resources. By integrating genetic diversity analysis, this research provides scientific support for distinguishing the quality of *M. basjoo* rhizomes from various origins and preventing market adulteration.

## 2 Materials and Methods

### 2.1 Experimental Materials

Forty‐five *Musa* spp. germplasms were collected from eight counties in Guizhou, Guangxi, Jiangxi, Jiangsu, and Fujian provinces, all of which are major production areas for *Rhizoma Musae*. Fresh, healthy leaves were collected and stored in paper molecular sample collection bags, which were subsequently placed in sealed boxes containing dry silica gel for preservation prior to analysis. Detailed sampling information was provided in Table 1. The samples were identified on the basis of botanical taxonomic criteria by Han Shuquan, Associate Researcher of Guizhou Institute of Subtropical Crops.

**TABLE 1:** Location information of 45 *Musa* spp. samples.

No.	Species	Classification	Sampling location	Province
GX (1–5)	*Musa basjoo*	Wild populations	Lingshan County, Qinzhou City	Guangxi Zhuang Autonomous Region
JX (1–5)	*Musa basjoo*	Wild populations	Yifeng County, Yichun City	Jiangxi Province
JS (1–5)	*Musa basjoo*	Wild populations	Shuyang County, Suqian City	Jiangsu Province
FJ (1–5)	*Musa basjoo*	Wild populations	Guangze County, Nanping City	Fujian Province
GZ (1–5)	*Musa basjoo*	Wild populations	Tianzhu County, Qiandongnan Prefecture	Guizhou Province
WM‐1	*Musa basjoo*	Wild populations	Qianxinan Bouyei and Miao Autonomous Prefecture	
WM (2–5)	*Musa nana*	Cultivated populations		
WM (6–11)	*Musa balbisiana*	Wild populations		
WM (12–19)	*Musa nana*	Cultivated populations		
SQ (1)	*Musa basjoo*	Wild populations	Shiqian County, Tongren City	

### 2.2 Transcriptome Sequencing

For RNA extraction and cDNA library construction, fresh leaves were sampled from *M. basjoo* specimens cultivated in the Wangmo Science and Technology Demonstration Park of the Guizhou Institute of Subtropical Crops. Total RNA was extracted from *M. basjoo* samples using the Total RNA Extractor kit (Shanghai Sangon Biotech, China). RNA concentrations were determined using a NanoDrop 2000 (OD260/280 1.8–2.0), with qualified samples used for subsequent cDNA library construction. The mRNA sample was sequenced using the Illumina NovaSeq 6000 platform by Majorbio (Shanghai, China), generating high‐quality raw data with an error rate below 0.1%, Q20 > 85%, and Q30 > 80%. Clean reads were then assembled de novo using Trinity (https://github.com/trinityrnaseq/trinityrnaseq/wiki) to generate a transcriptome from EST sequences, which was further optimized to obtain a non‐redundant unigene set. The resulting Unigene was then compared against the GO (Gene Ontology), KEGG (Kyoto Encyclopedia of Genes and Genomes), EggNOG (Evolutionary Genealogy of Genes: Non‐supervised Orthologous Groups), NR (NCBI Non‐Redundant Protein Sequence Database), Swiss‐Prot (Swiss‐Prot Sequence Database), and Pfam (Protein Families Database) databases for annotation.

### 2.3 Primer Design and Synthesis

The *M. basjoo* EST‐SSR loci were identified using MISA (http://pgrc.ipk‐gatersleben.de/misa/misa.html, default parameters). Primer design was performed using Primer3 v2.3.4, yielding 100 primer pairs selected on the basis of criteria including ≥ 10 repeats of dinucleotide, removal of redundant sequences, and random selection of those with predicted amplicons > 110 bp. These primers exhibit lengths of 18–25 bp, GC contents of 40%–60%, annealing temperatures of 55°C–65°C, and expected amplicon sizes of 100–300 bp.

### 2.4 SSR Polymorphic Primer Screening

Hundred mg of freeze‐dried leaves from each sample was used with the DNAsecure Plant Kit (TIANGEN, Beijing) for genomic DNA extraction. Four *Musa* spp. germplasms exhibiting significant phenotypic variation were selected for SSR primer screening. The 20 μL reaction mixture consisted of 2 μL DNA template, 2× Rapid Taq Master Mix, 0.6 μL of each upstream and downstream primer (10 μmol/L), and 7.4 μL ddH_2_O. The PCR amplification protocol was as follows: initial denaturation at 94°C for 5 min; followed by denaturation at 94°C for 30 s, annealing at 60°C for 30 s, and extension at 72°C for 50 s for 10 cycles; then 27 cycles with denaturation at 94°C for 30 s, annealing at 54°C for 30 s, and extension at 72°C for 50 s; and a final extension at 72°C for 5 min, with storage at 4°C. PCR products were first analyzed by 1.5% agarose gel electrophoresis to confirm the absence of discrete bands. Subsequently, the samples were subjected to 10% polyacrylamide gel electrophoresis, from which 10 highly polymorphic primer pairs were selected.

### 2.5 Identification of *M. basjoo* Germplasms

DNA from 45 *M. basjoo* samples was subjected to PCR amplification following fluorescent labeling with 6‐carboxyfluorescein (FAM). Capillary electrophoresis, conducted on an ABI‐3730XL Genetic Analyzer (Applied Biosystems, Foster City, CA), enabled the detection of fluorescence signals and corresponding peak locations.

### 2.6 Genetic Diversity Analysis

Genetic diversity was analyzed using Genemarker V2.2.0 to process the raw data obtained via capillary electrophoresis. Fragment sizes were determined by comparing the molecular weight internal standard position in each lane with the corresponding peak of each sample. The genetic diversity for each primer and population was assessed with POPGENE32 software. Genetic differentiation between populations was quantified using Nei's genetic identity (I) and genetic distance (D) (Nei 1973), chosen for their compatibility with SSR allele frequency data and alignment with standard practices in germplasm studies, ensuring comparability with future marker development research in *Musa* spp. On the basis of genetic distance, the UPGMA tree was constructed in MEGA 11, followed by secondary modification on the iTOL platform (https://itol.embl.de/).

## 3 Results

### 3.1 Functional Annotation of *M. basjoo* Transcriptome Unigenes

High‐throughput transcriptome sequencing generated 7.65 Gb of high‐quality data, with Q20 and Q30 values of 99.07% and 96.83%, respectively, and a GC content of 51.21%, indicating robust sequencing quality suitable for further analysis. Sequence assembly was performed using Trinity software, resulting in 38,806 Unigenes with an average length of 993.00 bp. Comparative analysis of all genes and transcripts against six major databases (GO, KEGG, eggNOG, NR, Swiss‐Prot, and Pfam) (Table 2) yielded annotated Unigene counts of 23,558, 12,412, 25,448, 28,101, 20,056, and 18,460, respectively.

**TABLE 2:** Functional annotation of *Rhizoma Musae* transcriptome unigenes.

Databases	Unigene number	Percent
GO	23,558	60.71%
KEGG	12,412	31.98%
eggNOG	25,448	65.58%
Nr	28,101	72.41%
SwissProt	20,056	51.68%
Pfam	18,460	47.57%
Number of Unigene annotated in databases	28,261	72.83%
Total Unigene	38,806 (1)	100%

#### 3.1.1 Nr Functional Annotation

**FIGURE 1:**
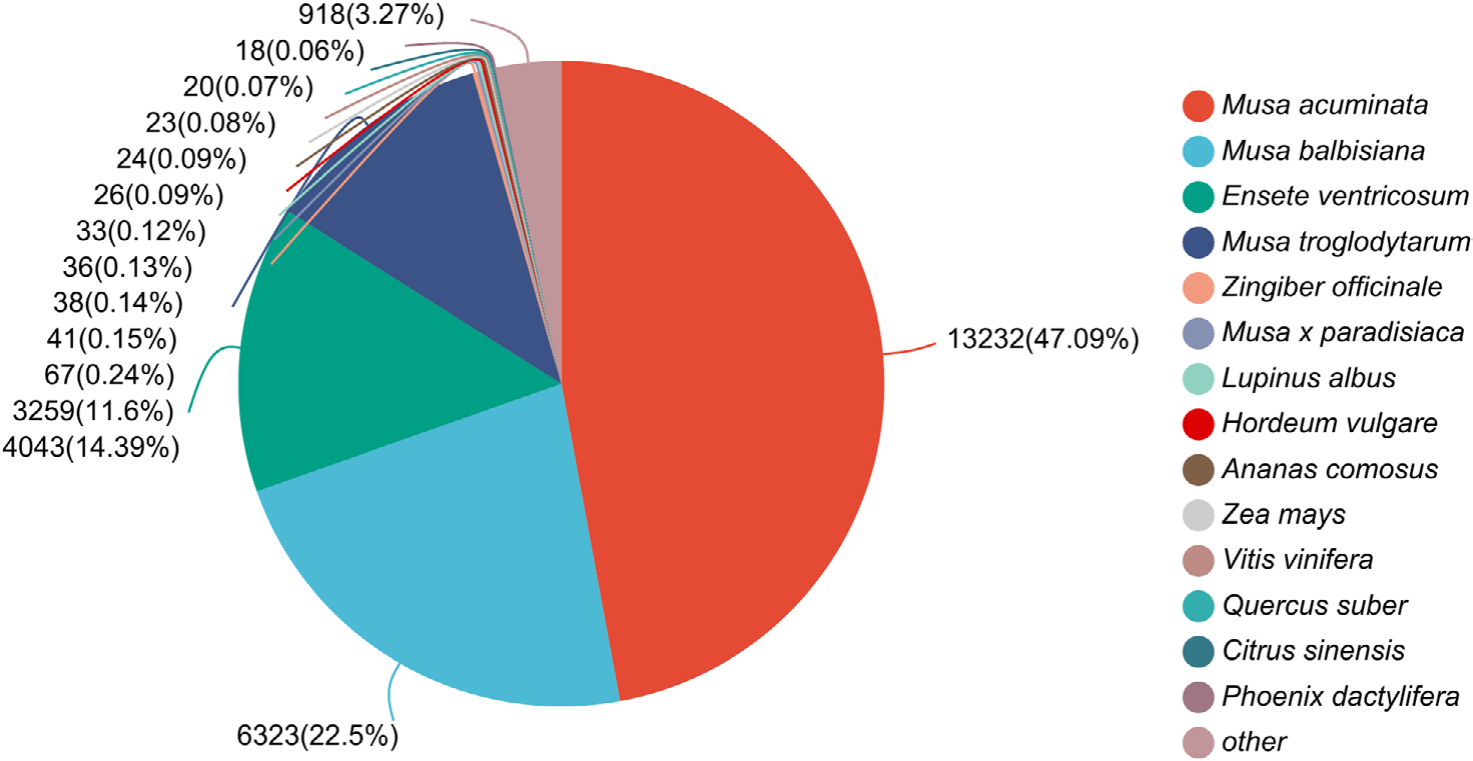
Nr functional annotation of *M. basjoo* transcriptome.

Comparison of the *M. basjoo* transcriptome with the Nr database revealed the following species with the highest sequence similarity: *Musa acuminata* (13,232 entries, 47.09%), *Musa balbisiana* (6323 entries, 22.50%), *Ensete ventricosum* (4043 entries, 14.39%), and *Musa troglodytarum* (3259 entries, 11.60%) (Figure 1).

#### 3.1.2 GO Functional Annotation

**FIGURE 2:**
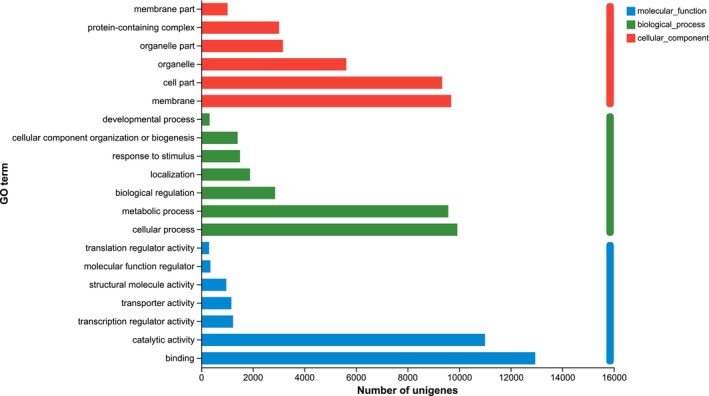
GO functional classification.

The Unigenes in the *M. basjoo* transcriptome were assigned to three GO categories: biological process (BP), cellular component (CC), and molecular function (MF), resulting in a total of 48 annotations (Figure 2). Among the 12 CC entries, the most annotated Unigenes were associated with membrane, cell part, and organelle. For BP, the 21 entries revealed predominant annotations in cellular process, metabolic process, and biological regulation. In the MF category, 15 entries were identified, with binding, catalytic activity, and transcription regulator activity being the most prevalent.

#### 3.1.3 KEGG Functional Annotation

**FIGURE 3:**
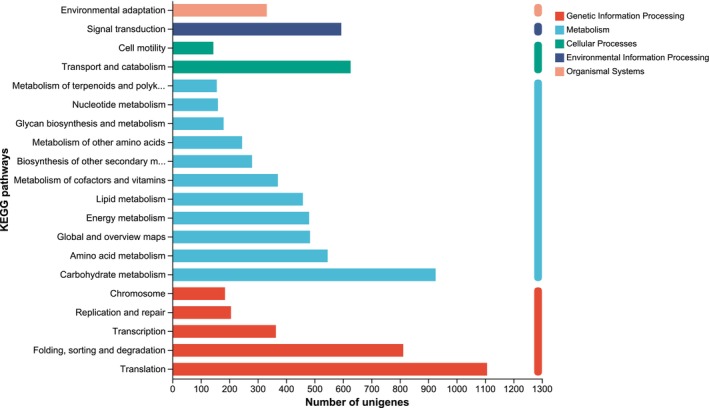
KEGG classification of *M. basjoo* transcriptome.

Unigenes from the *M. basjoo* transcriptome were mapped to 22 KEGG pathways across five primary categories: metabolism, genetic information processing, cellular processes, environmental information processing, and organismal systems (Figure 3). The largest number of Unigenes was assigned to the metabolism category, with a total of 4288 annotations. Within metabolism, carbohydrate metabolism accounted for the highest number of Unigenes (926), followed by amino acid metabolism (546), global and overview maps (484), and energy metabolism (481).

### 3.2 SSR Loci Information in *M. basjoo* Transcriptome Sequences

SSR loci in the *M. basjoo* transcriptome Unigene were analyzed using MISA software, with the results summarized in Table 3. A total of 7501 SSR loci were identified, yielding a distribution frequency of 19.33%. SSRs were classified into six categories on the basis of repeat type, with trinucleotide repeats constituting the majority (44.03%), followed by dinucleotides (43.87%), mononucleotides (24.73%), tetranucleotides (1.64%), hexanucleotides (0.52%), and pentanucleotides (0.51%). The most frequent repeating units were AG/CT, A/T, and ACC/GGT, representing 32.78%, 20.20%, and 2.41%, respectively. These findings establish a foundation for developing molecular markers specific to *Rhizoma Musae*.

**TABLE 3:** Distribution of SSR loci repeat numbers in *M. basjoo* transcriptome.

Repeat types	Number	Ratio of repeat (%)	The largest repeat unit	Percentage of the largest repeat unit (%)	Repeat number of SSR			
					1–5	6–10	11–15	> 15
Mononucleotide	1609	24.73	A/T	20.20%	0	733	789	87
Dinucleotide	2854	43.87	AG/CT	32.78%	0	1829	766	259
Trinucleotide	2864	44.03	ACC/GGT	2.41%	1394	1418	43	9
Tetranucleotide	107	1.64	ACAT/ATGT	0.05%	72	35	0	0
Pentanucleotide	33	0.51	CCCCG/CGGGG	0.01%	30	3	0	0
Hexanucleotide	34	0.52	AAGCAG/CTGCTT	0.01%	25	9	0	0
Total number	7501	100.00	—	55.47%	1521	4027	1598	355

### 3.3 EST‐SSR Primer Screening and Genetic Diversity Analysis

**FIGURE 4:**
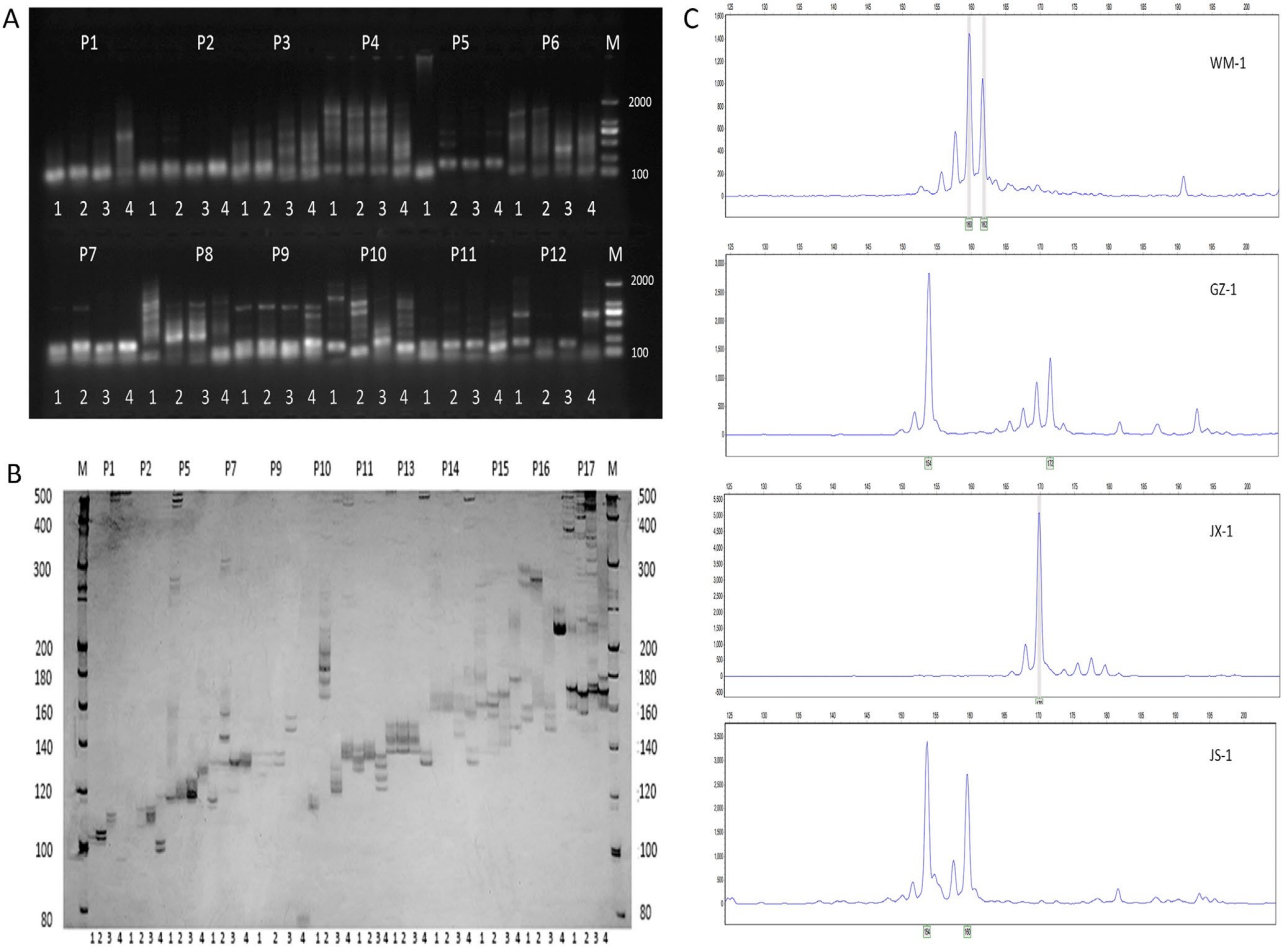
Partial results of EST‐SSR primer screening. (A) 1.5% agarose gel electrophoresis. (B) 10% polyacrylamide gel. 1: GZ‐1; 2: JS‐1; 3: WM‐1; 4: WM‐19. (C) Capillary electrophoresis of samples amplified with P15 primers.

Four *Musa* spp. germplasms (GZ‐1, JS‐1, WM‐1, and WM‐19), exhibiting significant phenotypic variation, were selected for the amplification of 100 pairs of random primers. The preliminary screening outcomes demonstrated that 60 pairs of primers were capable of successfully amplifying clear bands and boasted excellent repeatability, achieving an amplification success rate of 60% (Figure 4A). Subsequently, the 60 pairs of primers obtained from the initial screening were respectively analyzed through 10% polyacrylamide gel electrophoresis (with part of the results presented in Figure 4B). Eventually, 10 pairs of primers with remarkable polymorphism were screened out (refer to Table 4), with a primer effectiveness rate of 16.67%. Finally, the 10 pairs of polymorphic primers that had been selected were employed to carry out capillary electrophoresis amplification on 45 *Musa* spp. resources, and part of the results are presented in Figure 4C.

**TABLE 4:** Information on 10 EST‐SSR primer pairs.

Primer ID	Repeat motif	Primer sequence (5′‐3′)	Product length (bp)
P15	(GA)12	F: GTCATCTCGGTGTGCTGTGT R: TTGGTCTTGCAAGTGTCGAG	163
P17	(GA)15	F: CTTATCCGCTCCTTTTGTCG R: TTCCGATCGAGCAACTTCTT	172
P33	(GA)11	F: CAGGAAGGCAAATTCCAATC R: TCCTTCAAGATCTATGCCCG	193
P37	(TG)13	F: CAAGTCCCTTCGTAGCTCCA R: TGAGTTGTCGATCCTTGTCG	205
P49	(AC)11	F: TGGGTTGCATGAGCATTTTA R: GAGGAGCATTTATGCCCAAG	223
P57	(GA)23	F: GTCCCTCGTACCATCTCTCG R: TCCCCTCCCTTCTTTTTCTC	233
P62	(CT)10	F: ACTGATTTCGTCTCCATCGG R: AGGGATAAGCATCAAGCACG	237
P68	(GA)17	F: ACCAGCGCACAAGCAATACT R: CAAAGTGGTGGGAGACTGGT	245
P78	(TC)10	F: AAATTGCTAGTCCCACACGG R: ATACGCAACTGCTGCACATC	263
P92	(GA)11	F: TACAAATTCCGTTGGTCGGT R: GATGGCATGAAAACGATGTG	275

### 3.4 Genetic Diversity Analysis of 45 *Musa* spp. Germplasms

Genetic diversity analysis (Table 5) was conducted on 45 *Musa* spp. germplasms using the selected polymorphic primers, revealing a total of 104 alleles (Na), with an average of 10.400 alleles per primer pair. The number of effective alleles (Ne) ranged from 3.164 to 8.691, with an average of 5.195. The effective allelic variation proportion (Ne/Na) was 49.95%. Shannon's index (I) varied from 1.459 to 2.339, with an average of 1.835. Observed heterozygosity (Ho) ranged from 0.222 to 0.733, averaging 0.576, whereas expected heterozygosity (He) ranged from 0.692 to 0.895, with a mean of 0.795. Polymorphic information content (PIC) values ranged from 0.639 to 0.874, with an average of 0.759. The range of fixation index variation (Fis) was 0.112 to 0.698, with an average of 0.270. Overall, the 10 EST‐SSR primer pairs demonstrated high polymorphism, reflecting substantial genetic diversity across the 45 *Musa* spp. germplasms.

**TABLE 5:** Genetic diversity analysis of *Musa* spp. germplasms using 10 pairs EST‐SSR markers.

Primers	Na	Ne	I	Ho	He	PIC	Fis
P15	13	4.402	1.858	0.556	0.782	0.749	0.281
P17	13	5.745	1.992	0.733	0.835	0.805	0.112
P33	10	3.716	1.705	0.600	0.739	0.707	0.179
P37	7	3.799	1.492	0.222	0.745	0.693	0.698
P49	9	3.164	1.459	0.511	0.692	0.639	0.253
P57	14	8.691	2.339	0.533	0.895	0.874	0.397
P62	8	4.197	1.644	0.489	0.770	0.727	0.358
P68	13	8.385	2.280	0.711	0.891	0.869	0.193
P78	9	5.656	1.934	0.667	0.833	0.804	0.190
P92	8	4.197	1.644	0.733	0.770	0.729	0.037
Mean	10.400	5.195	1.835	0.576	0.795	0.759	0.270

Abbreviations: Fis, fixation index; He, expected heterozygosity; Ho, observed heterozygosity; I, Shannon's information index; Na, number of alleles; Ne, number of effective alleles; PIC, polymorphism information content.

**FIGURE 5:**
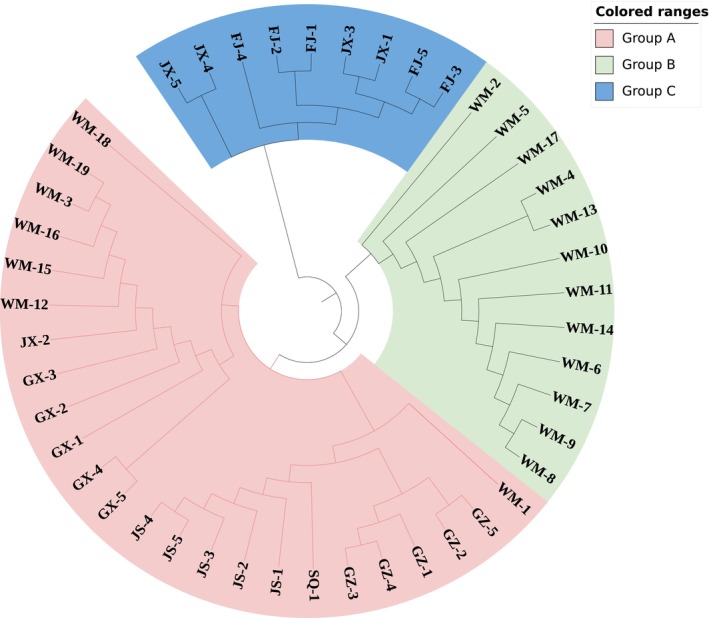
Phylogenetic tree of 45 *Musa* spp. germplasms using 10 EST‐SSR markers.

On the basis of the genotyping results obtained using 10 EST‐SSR markers, 45 *Musa* spp. germplasms were further analyzed by clustering using the UPGMA method. The UPGMA tree revealed three distinct clusters (Figure 5). Cluster A included 36 individuals from the GZ, JS, GX, and some WM populations; Cluster B comprised 12 individuals from the remaining WM population; and Cluster C contained 9 individuals from the FJ and JX populations.

## 4 Discussion

Recent advancements in transcriptome sequencing technology and decreasing sequencing costs have enabled the generation of transcriptome data, providing a valuable resource for developing molecular markers in medicinal plants lacking reference genomes. Pan et al. (2024) assembled 37,387 Unigenes and identified 11,021 SSR sequences from transcriptome data of *Polygonatum sibiricum* rhizomes, subsequently developing 21 polymorphic SSR markers across 24 *Polygonatum sibiricum* germplasms. Kapoor et al. (2023) performed transcriptome sequencing of *Phyllanthus emblica L. (Aonla)*, generating 87,771 Unigenes and designing 30 EST‐SSR primer sequences. Liu et al. (2021) identified 47 pairs of polymorphic SSR primers through transcriptome sequencing of *Cephalotaxus oliveri*. In *Musa* spp., Sampaio et al. (2024) developed banana germplasm resistant to key biotic and abiotic stresses using IRAP, ISSR, and SSR molecular marker technologies. Hinge et al. (2022) employed 34 molecular markers to establish a biomarker spectrum for banana identification. In this study, EST‐SSR markers were developed on the basis of *M. basjoo* transcriptome data, allowing preliminary provenance classification using origin information. The markers enable genetic differentiation of populations from distinct origins, facilitating targeted in situ conservation of regions with unique genetic profiles. Moreover, as the markers underpin the identification of *Rhizoma Musae* for Miao medicine, conservation can be strategically focused on safeguarding the genetic resources essential for maintaining the medicinal quality and ensuring the long‐term availability of this valuable herbal material.

Transcriptome sequencing of *M. basjoo* identified 38,806 Unigenes with annotation data. Comparative analysis of these Unigenes against the eggNOG database revealed 293 annotations related to the biosynthesis, transport, and catabolism of secondary metabolites. Further analysis with the KEGG database identified 280 Unigenes associated with the biosynthesis of various secondary metabolites, and 156 Unigenes linked to the metabolism of terpenes and polyketides. Previous studies indicate that *Rhizoma Musae* contains lupenone and β‐sitosterol, compounds capable of stimulating pancreatic β‐cell insulin secretion, enhancing glucose uptake and utilization, and ultimately reducing blood glucose levels (Xu et al. 2014). Moreover, 7beta‐hydroxyrutaecarpine, 7,8‐dihydroxycoumarin, and pinocembrin diacetate are among the crucial active components in *Rhizoma Musae* (Zhang et al. 2025b). They play roles in promoting osteocyte proliferation and exert anti‐inflammatory effects via signaling pathways such as the MAPK signaling pathway, Lipid and Atherosclerosis, PI3K‐Akt, and IL‐17. The Unigene annotation information of *M. basjoo* is anticipated to offer a reference for the dissection of the metabolic pathways of key active ingredients in *Rhizoma Musae* as well as the exploration of key genes.

When a species lacks reference genome information, leveraging high‐throughput transcriptome sequencing to develop SSR molecular markers is indeed a good approach, which features the advantages of low cost and high efficiency. In this study, a total of 7501 SSR loci were retrieved, and the distribution frequency of SSR loci was 19.33%, higher than that of medicinal plants like *Coptis chinensis* (13.72%) (Cui et al. 2024), *Houttuynia cordata* (7.51%) (Li et al. 2016), and *Polygonatum cyrtonema* (9.73%) (Chen et al. 2020). This indicates that the number of SSRs in the banana root transcriptome is relatively plentiful. The types of SSR motifs in *M. basjoo* transcriptome are diverse, with trinucleotide repeats and dinucleotide repeats being predominant, accounting for 44.03% and 43.87%, respectively. Their motif structures are similar to those of *Aquilaria sinensis* (Xiang et al. 2024), *Fritillaria cirrhosa* (Zhang and Li 2018), and *Andrographis paniculata* (Li et al. 2018). Metzgar et al. (2000) held that when SSR motifs that are not multiples of 3 are located in the coding region, they will trigger frameshift mutations, and this mutation pressure restricts the occurrence frequency of such SSR motifs. Haq et al. (2014) also discovered that trinucleotide repeats exhibit high abundance in the plant genome.

PIC is a significant genetic parameter. Botstein et al. (1980) reported that a PIC value greater than 0.5 indicates high polymorphism. In this study, the PIC values of the 10 pairs of EST‐SSR primers selected were all greater than 0.5, with an average PIC value of 0.759. Notably, these markers surpass the 12 SSR primers for *Musa* spp. developed by Hinge et al. (2022), which exhibited PIC values ranging from 0.00 to 0.37 (mean = 0.12), thereby demonstrating substantially stronger genetic discriminatory power. The UPGMA tree, on the basis of genetic distance, categorized the 45 *Musa* spp. germplasms into three groups. Specifically, the individuals from GZ (including SQ), JS, and GX were grouped into one category, whereas those from FJ and JX were clustered into another. This might be because the samples were collected from the wild, and because of geographical factors, there was relatively limited communication among them, thereby reflecting the differences among populations. This result is consistent with the analysis of Zhou et al. (2024). The individuals in the WM group were classified into two categories mainly because the samples were sourced from the germplasm resource nursery of this research institute, and the germplasm resources were collected from different geographical regions, thus resulting in different classifications. The classification results of the WM group individuals highlight the significant influence of geographical origin on germplasm characteristics. It further emphasizes the complexity and diversity within *Musa* spp. germplasm resources. However, the lack of controlled sampling years and cultivation history discrepancies in germplasm bank samples limit the functional interpretation of genetic clusters. Future studies could focus on exploring deeper into these regional differences to better understand the genetic variations and potential unique traits among different *Rhizoma Musae* germplasms, which will surely contribute to more comprehensive utilization and conservation of these valuable resources.

## 5 Conclusion

In summary, high‐throughput sequencing technology is employed to characterize the *M. basjoo* transcriptome, resulting in the identification of 38,806 Unigenes and the discovery of 7501 SSR loci, with an SSR locus distribution frequency of 19.33%. Among the repeat motifs, trinucleotide repeats are most prevalent, comprising 44.03%, with the dominant types being AG/CT, A/T, and ACC/GGT. Following polymorphism validation, 10 highly polymorphic and reproducible SSR markers were developed from the *M. basjoo* transcriptome database, capable of effectively distinguishing 45 *Musa* spp. germplasm resources. These SSR markers serve as a scientific basis for classifying the provenance of *M. basjoo* and identifying adulterants of its rhizomes. In the future, linking the medicinal components of *Rhizoma Musae* with molecular markers will facilitate the screening of high‐yielding *M. basjoo* resources.

## Author Contributions

**Jian Zhang:** conceptualization (lead), data curation (lead), funding acquisition (lead), investigation (lead), methodology (lead), resources (lead), writing – original draft (lead). **Chenglong Yang:** formal analysis (lead), methodology (lead), supervision (equal), writing – review and editing (equal). **Xiaoyu Yang:** investigation (supporting), methodology (supporting), writing – original draft (supporting), writing – review and editing (supporting). **Jing Lei:** methodology (supporting), validation (supporting), writing – review and editing (supporting). **Peiling Long:** investigation (supporting), writing – original draft (supporting).

## Conflicts of Interest

The authors declare no conflicts of interest.

## Data Availability

All relevant data for this study are publicly available from the NCBI database under BioProject PRJNA1213877 (https://dataview.ncbi.nlm.nih.gov/object/PRJNA1213877).
